# Indications for computed tomography (CT-) diagnostics in proximal humeral fractures: a comparative study of plain radiography and computed tomography

**DOI:** 10.1186/1471-2474-10-33

**Published:** 2009-04-02

**Authors:** Christian Bahrs, Bernd Rolauffs, Norbert P Südkamp, Hagen Schmal, Christoph Eingartner, Klaus Dietz, Philippe L Pereira, Kuno Weise, Erich Lingenfelter, Peter Helwig

**Affiliations:** 1 Klinik für Unfall- und Wiederherstellungschirurgie, BG-Unfallklinik Tübingen, Eberhard-Karls-Universität Tübingen, Schnarrenbergstr. 95, 72076 Tübingen, Germany; 2 Department für Orthopädie und Traumatologie, Albert-Ludwigs-Universität Freiburg, Hugstetter Str. 55, 79106 Freiburg, Germany; 3 Institut für Medizinische Biometrie, Eberhard-Karls-Universität Tübingen, Westbahnhofstr. 55, 72070 Tübingen, Germany; 4 Caritas-Krankenhaus Bad Mergentheim, Klinik für Unfall- und Wiederherstellungschirurgie, Uhlandstr. 7 97980 Bad Mergentheim, Germany; 5 Radiologische Klinik, Abt. für Diagnostische und Interventionelle Radiologie Universitätsklinikum Tübingen, Hoppe-Seyler-Straße 3, 72076 Tübingen, Germany; 6 Northland Bone and Joint Institute, 2750 Clay Edwards Dr. 304, Kansas City, MO 64116, USA

## Abstract

**Background:**

Precise indications for computed tomography (CT) in proximal humeral fractures are not established. The purpose of this study was a comparison of conventional radiographic views with different CT reconstructions with 2 D and 3 D imaging to establish indications for additional CT diagnostics depending on the fractured parts.

**Methods:**

In a prospective diagnostic study in two level 1 trauma centers, 44 patients with proximal humeral fractures were diagnosed with conventional X-rays (22 AP + axillary views, 22 AP + scapular Y-views) and CT (multi-planar reconstruction (MPR) and maximum intensity projection (MIP)) with 2 D and 3 D imaging. 3 observers assessed the technical image quality, the assessment of the relevant anatomical structures (2-sample-t-test) and the percentage of the osseous overlap of the proximal humerus (Welch-test) using a scoring system. The quality of the different diagnostic methods was assessed according to the number of fractured parts (Bonferroni-Holm adjustment).

**Results:**

There was significantly more overlap of the fractured region on the scapular Y-views (mean 71.5%, range 45–90%) than on axillary views (mean 56.2%, range 10.5–100%). CT-diagnostics allowed a significantly better assessment of the relevant structures than conventional diagnostics (p < 0.05) independently of the fracture severity (two-, three-, and four-part fractures).

**Conclusion:**

Conventional X-rays with AP view and a high-quality axillary view are useful for primary diagnostics of the fracture and often but not always show a clear presentation of the relevant bony structures such as both tuberosities, the glenoid and humeral head. CT with thin slices technology and additional 3 D imaging provides always a clear presentation of the fractured region. Clinically, a CT should be performed – independently of the number of fractured parts – when the proximal humerus and the shoulder joint are not presented with sufficient X-ray-quality to establish a treatment plan.

## Background

After physical examination plain X-rays are essential for diagnostic evaluation of proximal humeral fractures. High-quality radiological diagnostics is based on correct exposure, presentation of the shoulder joint in two views perpendicular to each other with minimal overlap of the fractured region by surrounding osseous structures and soft tissue [1]. Only if these requirements are met, an adequate evaluation of the individual topography, severity and direction of displacement of the fracture is possible, and a reliable classification of the fracture can be performed.

In particular, a computed tomography (CT) is recommended for complex fracture situations although those situations were not clearly defined. Therefore, precise indications for CT in proximal humeral fractures are not established. In addition, its benefit remains unclear [2-6].

A review of the literature showed that most often the anteroposterior view (AP view) and the scapular Y- view and the axillary view are used for routine diagnostics [7-11].

Various modifications of the axillary view of the shoulder are described [12-15]. The shoulder joint lies between the sagittal and coronal plane of the body and therefore correct radiological presentation is difficult[16]. Interpretation of the X-rays is impaired by multiple fracture lines of the usually complex injury[5]. The purpose of this study was a comparison of conventional radiographic views (AP view, scapular Y-view, axillary view) and the CT diagnostics (using either MIP or MPR reconstructions) in two-dimensional (2 D) and three-dimensional (3 D) visualization with either on film or individual on-screen presentation. Special attention was given to the technical quality of the method, osseous overlapping and assessment of relevant anatomical structures of the shoulder joint in proximal humeral fractures. We also wanted to establish precise indications for CT diagnostics depending on the part analysis according to the Neer classification.

## Methods

The study was performed with IRB approval in two university trauma centers. In this prospective study a consecutive series of 44 patients with acute proximal fractures that presented to the two university centers were included. No patients were excluded during this time period from the study.

In the first center 22 patients with a proximal humeral fracture (16 female/6 male, median age 64 years, range 36–94 years) were diagnosed with conventional analogous plain radiography with an AP view (66–70 kV/12.5–16 mAs) with the patient standing and the arm extended and an axillary view (66–70 KV/5–8 mAs) with the patient sitting and a minimum of 60° abduction of the arm in analogous technology and automatic exposure (Polydoros 50 s^®^, Siemens, Erlangen, Germany). Afterwards a standardized CT with the patient supine (Somatom Sensation 16^®^, Siemens, Erlangen, Germany) with MPR reconstruction of the data set and 2 D and 3 D imaging was performed. 2 D CT was carried out with a slice thickness of 0.75 mm in the osseous window. The 3 D reconstruction was performed with 1 mm layers. Films with 60 images for the 2 D CT and 12–15 pictures for 3 D reconstructions were printed and provided for viewing.

In the second center, for 22 patients with a proximal humeral fracture (17 women/5 men, median age 73 years, range 36–84 years), conventional digital radiography was applied with an AP view and a scapular Y-view with the patient standing (Device Polydoros Sx 50^®^, Siemens, Erlangen, Germany, Tube Optilix 150/30/50 C, Memory Foils System PCR Eleva Philips Electronics^®^, Hamburg, Germany).

Then, a 2 D and 3 D presentation after MIP reconstruction of the dataset (Siemens sensation 64^®^, Erlangen, Germany) with automatic individual adaptation was provided. Utilizing special software equipment, it was possible to assess each section of the CT-scans individually and from different directions with on-screen workstations. 2 D CT was carried out with a slice thickness of 0.75 mm in the osseous window. The 3 D reconstruction was performed with 1 mm layers. In general, 60 pictures for the 2 D CT and 12–15 for the 3 D-reconstructions were analyzed. The different radiographic views and CT techniques, different viewing methods, and different processing methods were used at each center, and therefore each center was considered as a separate group and both centers were compared with each other. Nevertheless, the consistencies of some AP views of the two centers were analyzed and were comparable.

All conventional X-rays were collected, scanned and analyzed with a processing program AutoCAD 2000^® ^(Autodesk GmbH Munich, Germany). According to the literature we defined the area of the proximal humerus as the square of the longest diameter of the epiphysis[17].

The percentage of the overlap surface of the proximal humerus by the surrounding osseous structures (acromion, lateral clavicula, coracoid, glenoid) were calculated and documented for the AP view, scapular Y- and axillary views.

Because of multi-planar visualization of the proximal humerus, there was no overlap in CT diagnostics.

All cases were presented to three investigators who were not involved in the care of the patients. Observer 1 was an experienced orthopaedic surgeon with fellowship training in shoulder surgery. Observer 2 was an experienced trauma surgeon with a special interest in bone and joint orthopaedic surgery. Observer 3 was a fellow for trauma surgery with a special interest in bone and joint surgery. The investigators independently evaluated the three imaging methods (conventional X-ray, 2 D CT, 3 D CT) for all 44 patients. All available images were used for evaluation of the relevant structures. They scored the technical quality of each of the two views of the conventional X-rays and the CT-scans separately. According to an imaging analysis score of Leschka et. al. and the European guidelines on quality criteria for diagnostic radiographic images we defined the essential parameters for the assessment as film density, contrast and sharpness of the relevant structures. The used scoring system consisted of 4 grades (1 = excellent, 2 = good, 3 = fair, and 4 = inadequate)[18,19]. For the assessment of the relevant structures the following five structures were defined to be relevant:

a) the greater tuberosity (on AP view), b) the glenoid and the humeral head (on AP view and axillary view/scapular Y-view), c) the coracoid (on axillary view/scapular Y-view), d) the lesser tuberosity (on axillary view/scapular Y-view) and e) the subacromial space (on AP view).

The essential parameters for the analysis of the relevant structures were the complete and clear presentation, the ability to estimate the degree of comminution and the degree of displacement. The scoring system consisted again of 4 grades (see above).

Average scores over all three investigators and two views were calculated for conventional and CT-diagnostics for assessment of technical quality and identifiability of relevant anatomical structures.

44 fractures were classified according to the part analysis of the Neer classification during a consensus conference of the three observers, who were not involved in patient care [20,21].

The statistical calculations were carried out with the statistics program JMP 6 (SAS campus drive, Building S., Cary, NC, 27513 SAS Institutes, Cary, NC, USA)

Frequencies of nominally scaled characters (part analysis of the Neer classification) were compared by a contingency table analysis with the chi square test. Averages of continuous variables were compared with the 2-sample-t-test, if the variance of the distributions did not differ significantly. For descriptive statistics we used the arithmetic means together with their 95% confidence intervals. For unequal variances the Welch-test was used. (E.g., proportion of overlap of the proximal humerus.) For multiple tests we used a Bonferroni-Holm adjustment of the significance level. For other statistical tests then the Bonferroni-Holm adjustment, the significance level was ρ < 0,05.

## Results

### Classification

In the first center there were twelve 2-part, seven 3-part and three 4-part fractures and in the second center there were nine 2-part, eight 3-part and five 4-part fractures. The whole group enclosed twenty-one 2-part, fifteen 3-part and eight 4-part fractures.

With regard to the part analysis of the Neer classification there were no significant differences between the centers. (ρ = 0.60)

### Technical image quality

**Figure 1:**
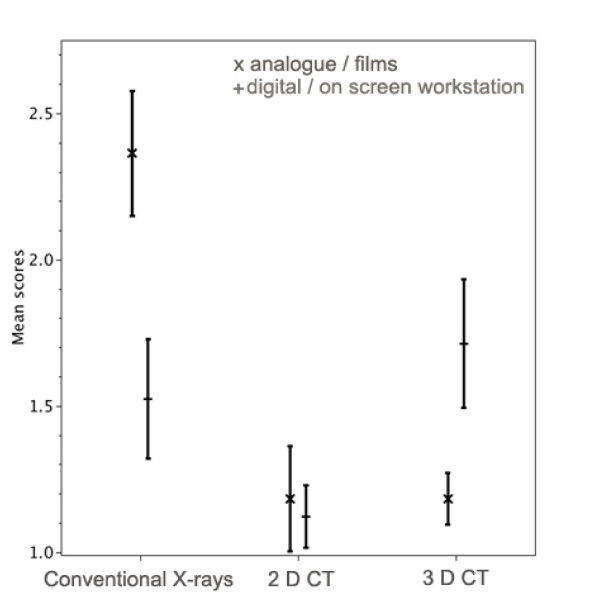
**Technical image quality**. The mean quality scores for the three methods (Conventional X-ray; 2 D CT, 3 D CT) and the two methods of presentations (x = analogue/films, + = digital/workstation) are shown with their 95% confidence intervals.

The mean quality scores for the three methods and the two centers are exhibited with their 95% confidence limits in Fig. 1. The quality of the conventional X-rays (AP and axillary views) carried out in analogous technology in center 1 was significantly worse than the X-rays made in digital technology (AP and scapular Y-view) in center 2. (ρ < 0.05) There was no significant difference in quality of X-rays made in digital technology and quality of 3 D CT images on a workstation.(ρ > 0.05) The quality of the 3 D CT images individually viewed on a workstation was significantly worse than the 3 D CT presented on films. (ρ < 0.05) There were no differences between the qualities of the 2 D CT techniques. (ρ > 0.05)

### Osseous overlap of the conventional X-ray diagnostics

**Figure 2:**
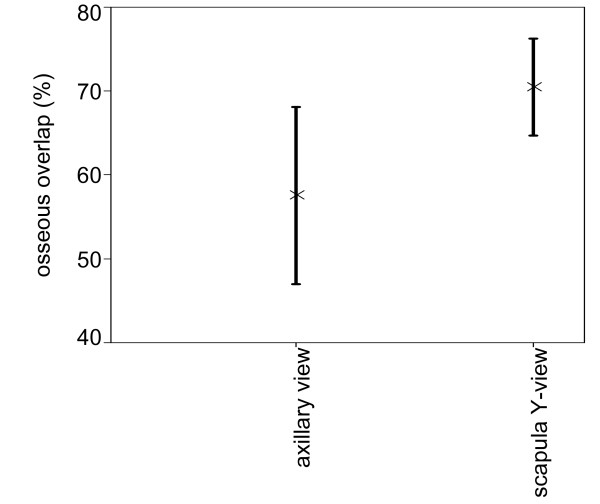
**Percentage of osseous overlap of conventional X-ray diagnostics (axillary view and scapular-Y-view) with their 95% confidence intervals**. Due to different variances in the two samples, the confidence intervals are different.

There was significantly more osseous overlap of the fractured region on the scapular Y-views than on axillary views.(ρ < 0.05) (Fig. 2). The scapular Y-views showed a median overlap of 71.5.% (range 45–90%). The median overlap of all axillary views was smaller with 56.2% but a much larger range (10.5–100%). The median overlap of the AP view was only 5.8% (range from 0 to 26%). For the AP view, there was no significant difference between the two centers (ρ = 0.7).

### Assessment of relevant structures of the shoulder joint and the proximal humerus

**Figure 3:**
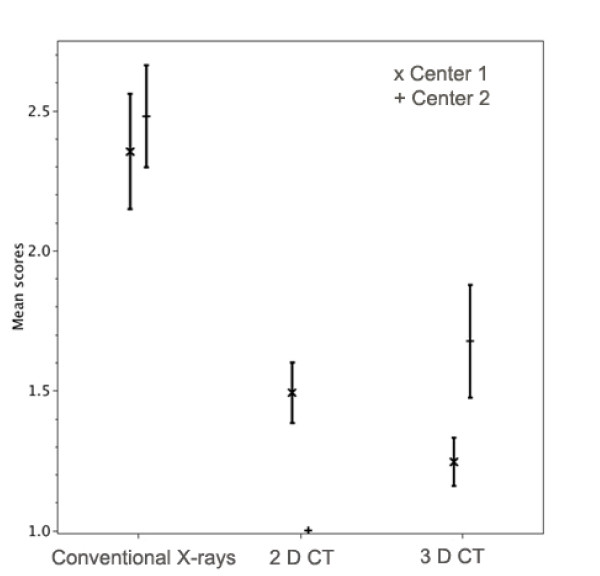
**Assessment of relevant structures of the shoulder joint and the proximal humerus**. The mean quality scores for the three methods and the two centers are shown with their 95% confidence intervals.

The relevant structures were graded significantly better on 2 D CT with individual visualization on a workstation than on 2 D CT presented on films (ρ < 0.05). 3 D viewed on films was significantly better than 3 D viewed on workstations. (ρ < 0.05) In addition, significant differences between the conventional radiographs and the 2 D CT and 3 D CT-investigations were found. (ρ < 0.05) (Fig 3)

### Neer classification for assessment of relevant structures of the shoulder joint and the proximal humerus

**Figure 4:**
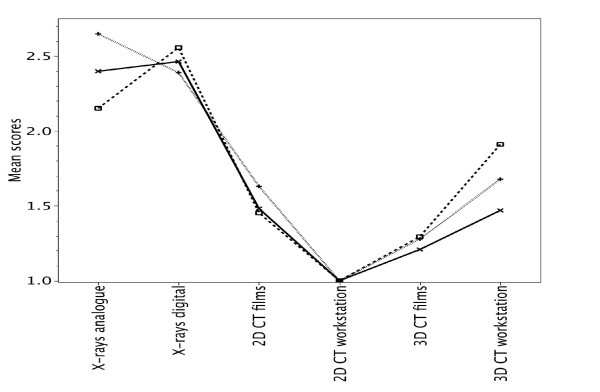
**Assessment of relevant structures of the shoulder joint and the proximal humerus according to fractured parts (2-parts = x, 3-parts = □, 4-parts = +)**. The mean quality scores for the three methods and the two centers are shown.

For all fracture severities – 2-, 3-, or 4-part fractures – and independently of the two centers, CT-diagnostics were significantly better than conventional radiographic diagnostics (ρ < 0.05). 2 D CT-diagnostics with image presentation on workstation were the best modality (Fig. 4)

## Discussion

A comparison of the different diagnostic methods with regard to technical quality, osseous overlapping and assessment of the relevant structures has not been presented in proximal humeral fractures. With the present investigation we tested various common conventional X-ray-views and CT in 2 D and 3 D imaging in proximal humeral fractures.

Our study showed a significantly better technical image quality of digital radiographs compared to analogous radiographs. In addition, 3 D CT reconstructions presented on films were better than 3 D CT reconstructions viewed on workstation. Furthermore, the digital conventional image quality was equal to the quality of the 3 D CT on workstation. Therefore, the quality standard, of the digital conventional radiography possibly was of such a high level that it reached the quality of a technically superior method such as 3 D CT. Those results may be related to a center-specific technical quality standard and the technical availability of modern diagnostic equipment and the expertise in digital image acquisition and processing.

Comparing the osseous overlap in different conventional radiographic views, our study showed that there was 15% less overlap of the fractured region by surrounding osseous structures on the axillary views than on the scapular Y-view. This resulted in a significant difference in the assessment of the relevant structures. Despite the importance of image quality, the choice of an appropriate view to minimize osseous overlap (e.g. axillary view) was found to be of equal importance for the visualization of the relevant structures.

The plain X-rays are still the most important tool for initial fracture diagnostics. In the commonly used trauma series the gold standard for initial evaluation is an AP. view in the plane of the scapula, a scapular Y- and the axillary view. According to Neer, for the initial evaluation of proximal humeral fractures an AP- view and a scapular Y-view are recommended. If the fracture visualization remains unclear, an axillary view is also necessary [21]. Our results demonstrate the superiority of the axillary view in overlap and the assessment of the relevant structures, when compared to the scapular Y-view. Similar to our study, other authors also recommend strictly the axillary view as a standard in combination with an AP view [11,22-24].

Sidor et al. classified 50 proximal humeral fractures with the help of the trauma series (AP, axillary, scapular Y-view). They examined the views of the trauma series according to their contribution of information for fracture classification. They showed that a correct classification of the fracture was possible by combination of AP view and axillary view in 99% of the cases. A combination of AP view and scapular Y-view resulted only in 79% of the cases in a correct classification. They concluded that an axillary view delivers significantly more information about fracture classification than the scapular Y-view [25].

The conduction of the AP view is usually not associated with technical problems. The AP view delivers an almost overlapping free visualization of the proximal humerus. Usually, an adequate assessment of relevant structures such as the greater tuberosity, glenoid and subacromial space is possible in most of the cases. Difficulties originate when X-rays are taken with the arm in internal rotation and when an inclination of the X-ray beam is chosen which is unsuitable to show the joint gap. Therefore, a posterior dislocation could be missed due to an overlap of the humeral head and the glenoid [23,26-28].

The scapular Y-view as a second view permits the assessment of the position of the joint and the relationship between the shaft and head of the humerus. However, we found an average osseous overlap of the proximal humerus and shoulder joint of about 70%. In addition, the evaluation of relevant structures, especially of the glenoid and the humeral head and the lesser tuberosity was considerably limited.

For the axillary view, an abduction of the arm is mandatory and therefore painful. The performance is technically demanding and difficult to reproduce. Although we have significantly better technical image quality in the digital than the analogous radiographs, we could show that the analogous axillary view of the fractured region resulted in significantly less osseous overlap of 56% than the digital scapula-y image (72%). Various modifications of this view were described [12-15]. However, it is common that the abduction may not be performed and the X-ray-evaluation may be limited. However, this was not a problem in the current study. Simon and co-authors evaluated axillary views on standardized osteotomized proximal humeri with different arm positions (in flexion, extension, abduction). They found, that in 30° of abduction of the arm, the real displacement of the fracture was not reproducibly represented on axillary views. Only if the arm is held in roughly 60° to 90° abduction in neutral position, reproducible results of the fracture displacement can be expected [29]. In our study all axillary views were performed with the patient sitting and 60–70° abduction of the arm with neutral rotation. With this approach a good assessment of the fractured proximal humerus, the head-shaft-axis, the position of the shoulder joint was possible and therefore adequate information was provided.

We could prove that even in simple fractures such as 2-part fractures according to Neer, the relevant bony structures of the humerus and shoulder joint were poorer to judge in the conventional X-rays than in the CT-scans. By multi-plane visualization of the fractured region in 2 D technology and 3 D-reconstruction of the dataset, the complete overlapping-free presentation is the most important advantage of this technique. All investigators graded the 2 D CT-scans as the best modality for diagnostics. In terms of technical image quality and presentation of the relevant structures, regardless of the fracture complexity, we found that the CT-diagnostics were better than the conventional X-rays. Various authors stated that the CT is a helpful diagnostic tool for evaluation of proximal humeral fractures. Nevertheless, those reviews included recommendations without exact definition of their use [2,3,30]. Because the visualization could be further improved by using a thinner layer thickness up to 0.75 mm, this method is now more often used for diagnostics in proximal humeral fractures. However, clear indications when to use a CT are not yet defined.

## Conclusion

If image quality impairs fracture visualization or if osseous overlap prevents the visualization of the fractured structures, conventional radiography is not sufficient. In such a situation, we believe that a CT should be performed.

## Competing interests

The authors declare that they have no competing interests.

## Authors' contributions

CB designed, coordinated and conceived the study, acquisition and analysis of data, wrote and corrected the manuscript. HS. participated in evaluation of radiographs and CT scans, corrected the manuscript and approved the final manuscript. EL participated in evaluation of radiographs and CT scans, corrected the manuscript and approved the final manuscript. BR coordinated and conceived the study, analysis of data, drafted the manuscript, revised the manuscript critically for important intellectual content. CE, NPS, PLP and KW revised the manuscript critically for important intellectual content and approved the final manuscript KD performed the statistical analysis, corrected the manuscript and approved the final manuscript. PH participated in evaluation of radiographs and CT scans, revised the manuscript critically and approved the final manuscript

## Pre-publication history

The pre-publication history for this paper can be accessed here:


